# Progressive painless lower limbs weakness in a dialyzed patient: undiagnosed tertiary syphilis: a case report

**DOI:** 10.1186/1757-1626-3-23

**Published:** 2010-01-13

**Authors:** O Dahmani, N Target, JP Guy, S Belkhelfa, F Mermet Jeanvoine, J Servonnat, J Djellid

**Affiliations:** 1 Department of nephrology, CH Louis Jaillon Saint-Claude, 39206, France; 2 Department emergency and trauma, CH Louis Jaillon Saint-Claude, 39206, France; 3 Department of Biochemistry and Microbiology, CH Louis Jaillon, Saint-Claude, 39206, France; 4 Department of internal medicine, CH Louis Jaillon Saint-Claude, 39206, France

## Abstract

**Introduction:**

Syphilis is a sexually transmitted disease, remaining under-estimated, under-recognized due to the variability of clinical presentation and ageing of the population with chronic comorbidities. Hence, some manifestations of the past are nowadays superimposed on the course of chronic diseases. Clinical suspicion should be guided by past medical history of contracting any other sexual disease in a heterosexual person or man who has sex with man.

**Case presentation:**

We describe a rare case of tertiary syphilis in a hemodialyzed diabetic patient whom was career of chronic liver disease due to the evolution of chronic hepatitis B virus infection complicated by a hepatocellular carcinoma. Initial orientation in diagnosing this rare presentation of progressive painless lower limbs weakness was attributed to possible side effects of ongoing anti viral therapy including lamivudine and adefovir. We continued administering both drugs while patient notified a spectacular improvement under Ceftriaxone therapy introduced empirically for a possible chest infection. Routine ophthalmologic examination realized in a teaching hospital, scheduled without knowing the course of late infection showed the presence of a syphilitic uveitis.

**Conclusion:**

This case emphasizes the need for a high index of clinical suspicion for syphilis before the occurrence of symptoms related to its end organ damage dominated by neurosyphilis form. Early diagnosis is the key to preventing significant morbidity and mortality and improving prognosis. However, in the setting of chronic diseases such as chronic kidney diseases either before setting up methods of renal replacement therapy or under immune-suppressive therapy; clinical presentation might resemble any disease, delaying the certitude of the diagnosis by prescribing a rapid plasma reagin.

## Introduction

Syphilis remains a common disease worldwide re-emerging as an important disease in western countries [1,2]. Untreated, during its latent phase; it could resemble any disease and escape from diagnosis unless having a high index of suspicion in a patient who contracted any other sexual transmitted diseases. Little is known about syphilis in dialyzed population. Herein, we report a hemodialyzed patient suffering from a progressive painless lower limbs weakness responding favourably to ceftriaxone therapy given initially for a possible chest infection.

## Case presentation

A 63-year-old married man of Asian ethnicity, on regular haemodialysis three times weekly undergoing diabetic nephropathy for the last two years, was complaining of having walking difficulties for two months. He was known to have chronic hepatitis B liver disease staged Child A with grade I oesophageal varices under anti viral therapy including the combination of lamivudine plus adefovir adjusted to kidney function tests. He also had a history of an operated hepatocellular carcinoma. He had many diabetic degenerative complications (proliferative retinopathy, stented ischemic heart disease, peripheral neuropathy) as well. He started complaining of a symmetrical painful proximal lower limbs weakness attributed initially to lumbar root compression according to lumbar CT scan and EMG/NCS findings. The former showed diffuse degenerative changes associated with a disc protrusion at L3-L4 level. The later showed evidence of a pending left external popliteal sciatic nerve without evidence of diffuse symmetrical polyneuropathy. Symptoms were relieved by benign pain killer and gabapentin 300 mg every other day. Three months later he arrived at the emergency department with mild fever, non productive cough, nocturnal sweat and inability to walk. Physical examination revealed an ill looking patient, weighing 63 kgs with blood pressure of 154/95 mmHg, heart rate 104 bpm regular, temperature 38.2 C and oxygen saturation of 95%. Glasgow score was 15 without meningismus or oto-ocular manifestations. Proprioception was conserved and Babinski, Hoffman and Laseque signs were all negative. The patient had mild walking abnormalities, MSGS at 3/5 and very poor squat test. However, both patellar reflexes were active, and symmetric. He had no pain or peripheral numbness. Cardiovascular examination showed moderate tachycardia without friction rub or murmurs. There were bi-basal crackles mostly on the right side with absence of dullness on percussion and hepatojugular reflux. The peripheral pulse was conserved without acrosyndrom. Skin examination showed pityriasis versicolor-like eruptions mainly on upper limbs and upper thorax. The external genitalia revealed hypochromic vitiligoid spots on the scrotum. He was free of lymphadenopathy. Results of laboratory investigations are shown in tables 1, 2 and 3. Sputum smear for bacterial and mycobacterium, urine C/S, blood C/S (3), Mantoux test, were all negative. iPTH = 205 ng/l (15-65), 1, 25(OH) D3 24 pmol/l (43 - 148) and 25 OH D3 = 38 nmol/l (75 - 200) with normal phosphocalcic product. VDRL = 2, TPHA 1280, FTA abs 100, FTA -M <10 and positive anti -TpN47, TmpA, TpN17 and TpN 15(IgG western-blot). EKG showed no new changes and transthoracic Doppler-echocardiography demonstrated normal left ventricular size and function apart from mild concentric hypertrophy. He had no significant valvulopathy, though a calcified bicuspid aortic valve with mild regurgitation was noted without Aortitis or aneurismal aspects. CXR disclosed an interstitial aspect of both field of the lungs and normal sized mediastinal silhouette. Funduscopic appearance showed beside proliferative retinopathy, a syphilitic uveitis thereafter, discovered incidentally elsewhere. Abdominal and pelvis ultrasound study demonstrated mild splenomegaly and poorly differentiated normal sized kidneys with acquired cystic disease. The patient received an empirical treatment of ceftriaxone 1.5 gr administered post-dialysis with a spectacular response within one week; therapy judged by net improvement of his weakness and return home walking. Ceftriaxone alone was maintained to complete duration of two weeks. Meanwhile, the spouse of the patient was convoked for screening. We learned that she had been receiving prednisolone and sub-cutaneous injection of infliximab weekly for the last six months, and ibuprofen for a relapsing rheumatoid arthritis. Awaited syphilitic serologic testing disclosed normal blood chemistry without inflammation (CRP = 5 mg/l). VDRL = 2, TPPA = 2560 FTA abs 200, FTA-M 20 (N <10). A medical report was sent to her general practitioner for intramuscular Benzathin-penicillin therapy 2.4 Millions units weekly over three consecutive weeks.

**Table 1:** Results of haematology laboratory tests

Variable	Patient	Normal range
Haemoglobin (gr/dl)	10	13 - 17
Hematocrit (%)	29.8	40 - 54
White cell count (per mm^3^)	10 000	4 000 - 10 000
Differential count:		
Neutrophils	8500	
Lymphocytes	760	
Monocytes	670	
Eosinophils	50	
basophils	20	
Platelets count (per mm^3^)	193000	150 000 - 350 000
Mean corpuscular volume(fl)	94.4	80 - 100
Prothrombin time (%)	91	70 - 100
International normalized ratio	1.07	
Partial thromboplastin time activated (sec); P/C	55/32	
Fibrinogen (gr/l)	5.6	2.5 - 4
D-dimmers (mcg/l)	0.8	0.5
CRP (mg/l)	48	< 5
Procalcitonin (mcg/l)	0.66	<0.5
Orosomucoid level (gr/l)	1.62	0.57 - 1.22
Haptoglobin (g/l)	2.79	0.87 - 2.32
Transferring (g/l)	2.04	1.77 - 2.83
Beta 2 microglobulin (mg/l)	10.40	1 - 2

gr/dl = gram per decilitre, mg/l = milligram per litre, mcg = microgram per litre, P/C = patient/control

**Table 2:** Results of blood chemistry tests

Variable	Patient	Normal range
Glucose (mmol/l)	7.1	3.1 - 6.4
HbA1c (%)	6.4	< 7 in diabetic patients
Urea nitrogen (mmol/l)	21.9	1.7 - 8.3
Creatinine (umol/l)	501	< 106
Calcium (mmo/l)	2.03	2.15 - 2.55
Phosphorus (mmol/l)	1.38	0.87 - 1.45
Sodium (mmol/l)	138	136 - 145
Potassium (mmo/l)	4.6	3.5 - 5.1
Chloride (mmol/l)	99	95 - 105
Carbon dioxide (mmol/l)	18	23 - 29
Total Bilirubin (umol/l)	10	< 22
Alanine aminotranferase	14	< 41 UI/l
Aspartate aminotransferase	46	< 37 UI/l
Alkaline phosphatise	902	< 270 UI/l
Gamma glutamyl transferase	476	< 49 UI/l
Lactate dehydrogenase	417	< 480 UI/l
Amylase	173	< 220 UI/l
Creatine kinase	113	< 190 UI/l
Lactic acid (mmol/l)	0.76	< 2
Iron (umol/l)	16.5	10.7 - 32
Iron binding capacity(umol/l)	46	45 - 72
Transferrin saturation coefficient (%)	0.36	0.15 - 40
Ferritin (ng/ml)	898.5	30 - 240
Ammonia (umol/l)	88	16 - 60
Plasma osmolarity (moSm/l)	315	275 - 305
Uric acid (umol/l)	276	< 420

Mmol/l = millimol per litre, μmol/l = micromol per litre, mosm/l = milliosmol per litre, ng/ml = nanogram per millilitre

**Table 3:** Results of immunology laboratory tests

Variable	Patient	Normal range
Antinuclear antibody (Abs)	< 1:80	> 1: 80
Anti DNA Abs	Negative	
Rheumatoid factor	Negative	
Antineutrophil cytoplasmic Abs		
Anti PR3 abs	< 10	< 20
Anti MPO abs	< 10	< 20
Complement		
Total	N/A	
C3	1.6	0.90 - 1.73
C4	N/A	
IgG	14.4	6.5 - 12.08
IgA	2.92	1 - 3.20
IgM	0.52	0.49 - 1.38
Cryoglobulin	NEGATIVE	
Anticardiolipin Abs	< 10	< 23
HCV Abs	negative	
HbsAg	positive	
HbsAbs	<5	
HbcAbs		
IgG	positive	
IgM	negative	
HIV 1 & 2	negative	
Thyroid function test	low T3 with normal TSH	
Chlamydia (Ig M)	< 10	< 10
Mycoplasma (Ig M)	negative	
Legionella urinary Ag	negative	
Lyme disease (IgM)	negative	
Plasma protein electrophoresis		
Total proteins(g/l)	74	66 - 87
Albumin (g/l)	37.4	36 - 48
Alpha 1 (g/l)	3	1 - 3
Alpha 2 (g/l)	11.4	4 - 8
Beta (g/l)	9.2	5 - 10
Gamma (g/l)	12.9	7 - 13
A/G ratio:	1.02	
Folic acid level (nmol/l)	9	> 7
B12 vitamin level (pmol)	477	133 - 675

Abs = antibodies, Ag = antigen, pmol/l = picomol per litre

## Discussion

The diagnosis of late latent syphilis is very difficult to make because of the diversity and similarity of its clinical presentation [1,3]. In fact, Sir William Osler stated that "syphilis simulates every other disease" owing to mixed and atypical forms. Besides having a high index of suspicion; past medical history of exposure to Treponema pallidum, and physical examination along with serologic tests remained paramount for the diagnosis. Cerebrospinal fluid (CSF) may be helpful, though neurosyphilis with normal CSF cell count in a HIV negative subject has been reported [4]. In addition a negative CSF VDRL cannot exclude it, and FTA-Abs is less specific, but more sensitive. In our case the diagnosis was difficult because of superimposed other diseases which unmasked its latent form by impairing immunity: complicated diabetes mellitus, Chronic HBV infection under anti-viral therapy, end stage renal failure and therapeutic agents prescribed for dialyzed patients. However, the diagnosis of tertiary syphilis has been guided by previous history of contracting sexually HbsAg and the slowly progressive proximal lower limbs weakness, associated with skin eruptions, pneumonitis and cholestatic hepatitis. Uveitis was diagnosed in another hospital without knowing the syphilitic course. Pulmonary involvement in our case filled the criteria proposed by Coleman et al [5], and ceftriaxone therapy was initially given empirically. The decision to continue ceftriaxone over two weeks was based on positive serologic tests. Shan et al [6], described a case of successful treatment of symptomatic neurosyphilis with ceftriaxone, as in ours. Echocardiography finding of calcified bicuspid Aortic valve might be a consequence of either syphilis, or secondary hyperparathyroidism, or both. The neurological signs and symptoms presented herein might evoke a picture similar to that of chronic myelopathy in which the pyramidal pathway is involved, characterized by active reflexes and positive Babinski's sign. This latter was negative probably due to peripheral polyneuropathy that was not confirmed by either physical examination or on EMG/NCS registering. A combined neuro-muscular syphilitic affection remained the most likely in addition to a painful radiculopathy abolished by pain killers. However, we can not exclude syphilitic spinal cord involvement by simple CT scan finding. EL Quessar et al [7], reported a case of spinal cord gumma with normal myelography and Brain CT. We didn't perform lumbar puncture or further explorations due to rapid clinical improvement under ceftriaxone therapy and antiviral drugs have been continued.

## Conclusion

Syphilis remains a great imitator because its signs and symptoms are not pathognomonic when superimposed upon chronic diseases yielding high morbidity. Keeping high grade of suspicion of syphilis in individual who are at risk of other sexually transmitted infection and vice-versa is mandatory to limit syphilitic end organ damage. In hemodialyzed patients ceftriaxone is gaining ground when treating acquired syphilis and is equivalent to benzathin penicillin not only because of its practical administration but due to impaired pharmacokinetics of the latter. The rule in treating the sex partner of an infected patient regardless of whether or not infected should be respected as in ours.

## Abbreviations

EMG/NCS: electomyogram test/nerve conduction studies; MSGS: muscle straight grading scale; VDRL: venereal disease reference laboratory; TPHA: treponema pallidum haemagglutination assay; FTA abs: florescent treponemal antibody-absorption test; C/S: culture and sensitivity; CXR: chest x-ray; EKG: electrocardiogram; iPTH: intact parathyroid hormone; HBV: hepatitis B virus; HCV: hepatitis C virus.

## Consent

Written informed consent was obtained from the patient for submission of manuscript for publication of this case. A copy of the consent is available for review by the Editor-in-Chief of this journal.

## Competing interests

The authors declare that they have no competing interests.

## Authors' contributions

All authors contributed to each stage of this work, OD, NT, JPG, SB, FMJ, JS and JD all have: (1) made substantial contributions to conception and design, or acquisition of data, or analysis and interpretation of data; (2) been involved in drafting and revising the manuscript; and (3) given final approval of the version to be published.
